# Is extended resistance to the historical antiretroviral drugs & drug classes still a risk factor for HIV progression?

**DOI:** 10.1186/1758-2652-13-S4-P138

**Published:** 2010-11-08

**Authors:** M Zaccarelli, P Lorenzini, P Marconi, F Forbici, C Gori, P Sette, F Ceccherini-Silberstein, P Narciso, CF Perno, A Antinori

**Affiliations:** 1 National Institute for Infectious Diseases “Lazzaro Spallanzani”, Clinical Department, Rome, Italy; 2 National Institute for Infectious Diseases “Lazzaro Spallanzani”, Virological Department, Rome, Italy; 3 University of “Tor Vergata”, Department of Experimental Medicine, Rome, Italy

## Background

Since recent observations demonstrated that extended resistance to all the three main antiretroviral classes (NRTI, NNRTI, PI) is a marker of disease progression and death, the aim of the present analysis is to evaluate if this situations persists in recent years when several new potent drugs entered in the current clinical use.

## Methods

Patients undergoing genotypic resistance test after treatment failure between 1999-2008 were included. The risk of progression was calculated with survival analysis separately for patients who failed between 1999-2003 and 2004-2008. Class resistance for the three historical drug classes was assessed, using Rega interpretation system (v. 8.0.1), when no fully active drug in each class was detected. Tipranavir, darunavir and etravirine were not included in the historical drugs classes. The follow-up was carried out up to December 2009: new AIDS event/death were considered study endpoint.

## Results

Overall, 1522 patients were included, of whom 782 in the 1999-2003 and 740 in the 2004-2008 group. During follow-up, 171 and 59 new AIDS/death events were observed in the two groups, respectively. At survival analysis, the proportion of patients who achieved the study endpoint after 5 year of observation was 24% in the 1999-2003 and 11% in the 2004-2008 group. In the 1999-2003 group, a higher risk of progression in patients with no active drug in all the three historical classes was found (41% vs. 19% in patients with ≥1 active class, p=0.03 at adjusted Cox model). In the 2003-2008 group, the risk of progression was lower in patients with 3-class resistance (25%) while less risk reduction was found in patients with ≥1 active class (14%). Indeed, in the 2003-2008 group, 67% of patients with 3-class resistance were treated with ≥1 drug among tipranavir, enfuvirtide, darunavir, raltegravir, maraviroc and etravirine, compared with 45% of patients with 2-class and 6% of ≤1-class resistance. The most widely used drug were darunavir (58% of 3-class resistant patients), tipranavir (55%) and enfuvirtide (45%).

## Conclusions

The availability for current use of new drugs, in new classes and those belonging to old classes but with different resistance patterns, may explain the improved survival of the more virologically impaired HIV patients. However, the improvement in survival does not still appear so crucial, particularly in patients with active drugs where a 14% progression at 5 years is still observed.

